# A novel solid beverage RLMCR prevents obesity-related metabolic disorders through the gut-adipose axis

**DOI:** 10.3389/fnut.2026.1895261

**Published:** 2026-07-30

**Authors:** Ying Wen, Yan-Mei Peng, Xuan-Yu Zhou, Hui-Xuan Wu, Chen-Yi Tang, Fei Cheng, Yu-Xin Han, Hong-Li Jiang, Zhuo Gong, Yan-Hong Bu, Long Li, Fen Xiao, Jun-Min Cai, Yu-Yao Mo, Qian Yang, Heng-Le Chen, Cong-Ying Zhan, Hou-De Zhou

**Affiliations:** 1 National Clinical Research Center for Endocrine and Metabolic Diseases, Hunan Provincial Key Laboratory for Metabolic Bone Diseases, and Department of Metabolism and Endocrinology, The Second Xiangya Hospital of Central South University, Changsha, Hunan, China; 2 Institute of Chinese Materia Medica, Hunan Academy of Chinese Medicine, Changsha, China; 3 Faculty of Medicine, Macau University of Science and Technology, Macau, China; 4 Department of Clinical Nutrition, The Third Xiangya Hospital of Central South University, Changsha, Hunan, China; 5 School of Public Health, Changsha Medical University, Changsha, Hunan, China; 6 Department of Blood Transfusion, The Second Xiangya Hospital, Central South University, Changsha, Hunan, China

**Keywords:** Akkermansia, gut-adipose axis, obesity, RLMCR, white adipose tissue browning

## Abstract

**Background:**

The global obesity epidemic and associated metabolic comorbidities have imposed an insurmountable strain on healthcare systems, underscoring the urgent need for simple, early interventions to curb obesity at its onset. To address this problem, we developed a novel solid beverage RLMCR (Chinese National Invention Patent No. ZL 202410541939.2) and proved its therapeutic effects on established obesity, yet its preventive potential and underlying mechanisms remain unelucidated.

**Purpose:**

This study aimed to further explore the preventive role of RLMCR on diet-induced obesity and elucidate its mechanism via the gut-adipose axis.

**Methods:**

Male C57BL/6J mice were given RLMCR throughout high-fat diet (HFD) feeding to assess its preventive efficacy against obesity. Post-intervention, key metabolic indices, including body weight, fat percentage, and glucose tolerance, were measured. Subsequently, energy expenditure was monitored, and cold tolerance assays, adipose histomorphology, and thermogenic gene expression analysis were conducted. To characterize the modulatory effects of RLMCR on gut microbiota, 16S rDNA sequencing and untargeted metabolomics analysis were performed. Fecal microbiota transplantation (FMT) was finally conducted to verify the mediating role of gut microbiota in the beneficial effects of RLMCR.

**Results:**

RLMCR markedly mitigated HFD-induced body weight gain and glucolipid metabolic disturbances, while significantly augmenting energy expenditure and cold tolerance. Besides, RLMCR facilitated white adipose tissue browning, as reflected by the emergence of multilocular adipocytes and the upregulation of core thermogenic genes including UCP1 and PGC-1α. Moreover, RLMCR effectively restored the HFD-induced gut microbial disruption and notably enriched the abundance of *Akkermansia*. FMT from RLMCR-treated donor mice recapitulated the enhanced adipose browning, thereby replicating the metabolic benefits of RLMCR in recipient mice.

**Conclusion:**

RLMCR effectively prevented HFD-induced obesity and metabolic dysfunction via gut microbiota-mediated adipose browning. These findings offer a novel preventive strategy for obesity and support the development of RLMCR-based anti-obesity products.

## Graphic abstract

**Figure:**
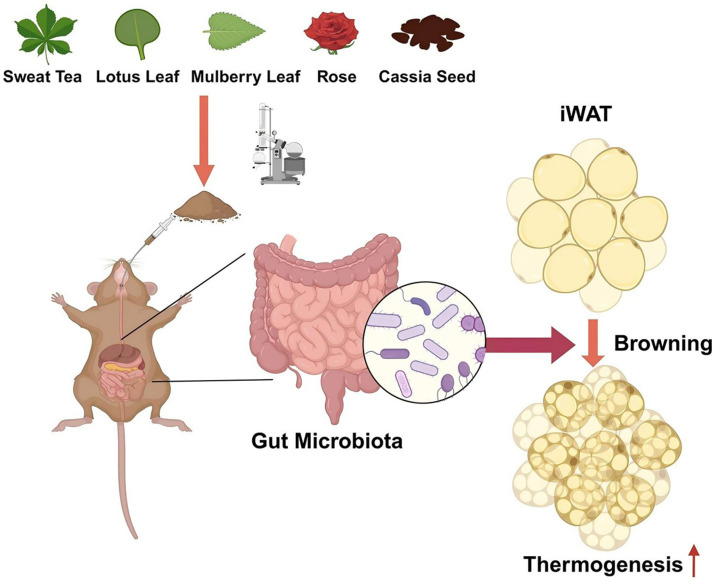
The novel patented solid drink RLMCR alleviates high-fat diet (HFD)-induced weight gain and metabolic disorders by regulating gut microbiota dysbiosis and inducing white adipose tissue (WAT) browning via the gut-adipose axis (created in BioRender).

## 1 Introduction

Obesity has become a global public health pandemic, affecting more than 1 billion people worldwide. Current projections show that by 2035, over 4 billion people—nearly half of the global population—will be affected by overweight or obesity (1). Importantly, the health risks of obesity go far beyond excess body fat accumulation. As a major established risk factor, rising obesity rates drive higher incidence of life-threatening chronic diseases, including type 2 diabetes, hypertension, and multiple cancers, placing a heavy dual burden on individual health and global healthcare systems (2). In fact, effective obesity control requires a combination of prevention and treatment: prevention targets early obesity onset, while treatment targets weight management and complications alleviation in existing patients. However, notable shortcomings currently exist in these two aspects. From the perspective of prevention, with rapid socioeconomic development and continuous advancement of health education, public awareness of obesity prevention has increased significantly, yet the absence of guideline-recommended preventive drugs leads to a gap between growing public health aspirations and limited preventive strategies. As for treatment, although therapeutic options for obesity are diverse, current regimens often carry significant adverse effects and fail to target multiple comorbidities simultaneously. Therefore, there is an urgent need to develop safe, multi-target interventions that integrate both preventive and therapeutic effects against obesity and its associated metabolic disorders.

To address the aforementioned issues, we innovatively developed a novel functional solid drink named RLMCR. This beverage consists of medicinal and edible plants, including *Rubus suavissimus* S. Lee (Chinese sweet tea), Lotus leaf, Mulberry leaf, *Cassia Seed*, and *Rosa rugosa* Thunb, all with proven weight-loss, hypoglycemic, and lipid-regulating properties. Under the guidance of the synergistic theory of Traditional Chinese Medicine, RLMCR was designed as a multi-target agent for obesity and its metabolic complications control, and has been granted a Chinese national invention patent (Patent No. ZL 202410541939.2). In our previous work, the active constituents of RLMCR were characterized by liquid chromatography–tandem mass spectrometry (LC–MS/MS), identifying flavonoids, phenolic acids, and alkaloids as its major components. Preliminary studies also verified its therapeutic efficacy in alleviating body weight gain and improving glycolipid metabolism disorders in diet-induced obese (DIO) mice (3). Nevertheless, the precise mechanisms underlying its beneficial effects remain unclear, and its preventive potential against HFD-induced obesity requires further systematic investigation.

Multiple factors contribute to the development of obesity, among which gut microbiota plays a pivotal role. Compared to germ-free mice, conventional mice are more susceptible to high-fat diet (HFD)-induced obesity and glucose metabolism disorders (4). Meanwhile, the gut microbial structure differs significantly between obese and lean individuals, which is marked by elevated *Firmicutes* and reduced *Bacteroidetes* abundance—a pattern consistently validated in both human and mouse models (5, 6). Adjusting this microbial structural dysbiosis can effectively alleviate obesity-related metabolic abnormalities, making gut microbiota modulation a promising target for obesity intervention (7). Emerging evidence indicates that medicinal and edible herbs exert prebiotic-like effects via diverse bioactive metabolites (e.g., polysaccharides, alkaloids, glycosides), reversing microbial imbalance in obesity and type 2 diabetes (8, 9). Consistent with this, the core ingredients of RLMCR—including *Rubus suavissimus* S. Lee, lotus leaf, mulberry leaf, and cassia seed—along with their active metabolites, have been proven to regulate gut microbiota structure (10–13). Consequently, we hypothesize that regulation of the gut microbial community represents a potential mechanism underlying the anti-obesity effects of RLMCR.

In our previous study, we only characterized the therapeutic effects of RLMCR against obesity in DIO mice, yet its molecular mechanisms remained unelucidated, hindering translational progress of this functional solid beverage. In fact, preventing the onset of obesity is critical to addressing this pervasive public health challenge, and targeted preventive interventions can resolve key limitations of conventional obesity management. In the present study, the preventive effects of RLMCR against HFD-induced obesity and metabolic dysregulation were validated for the first time. Systematic mechanistic exploration was conducted through 16S rDNA sequencing, untargeted metabolomics, and fecal microbiota transplantation (FMT) experiments. Our results demonstrated that the anti-obesity effect of RLMCR is mediated through the gut–adipose axis, which provides novel mechanistic insights into its biological functions. Collectively, this work capitalizes on the unique advantages of functional foods for preemptive disease intervention, proposes an original intervention strategy for obesity control, and establishes theoretical foundations for the broader clinical translation of RLMCR.

## 2 Materials and methods

### 2.1 Chemicals and reagents

The novel solid drink RLMCR consists of five medicinal and edible plants: *Rubus suavissimus* S. Lee, lotus leaves, mulberry leaves, cassia seed, and *Rosa rugosa* Thunb. *Rubus suavissimus* S. Lee was provided by Yijianing Science & Technology Co., Ltd. (Hunan, China). Lotus leaves, mulberry leaves, cassia seed, and *Rosa rugosa* Thunb were purchased from Zhenxing Chinese Medicine Co., Ltd. (Hunan, China). All raw materials were authenticated and quality controlled by specialists at the Hunan Academy of Chinese Medicine following the standards specified in the Chinese Pharmacopoeia. Voucher specimens were kept at the Institute of Chinese Materia Medica, Hunan Academy of Chinese Medicine (Voucher number: 20230131). The preparation method for RLMCR was identical to that described in our previous publication (3). Briefly, the five ingredients of RLMCR (sweet tea, lotus leaves, mulberry leaves, Cassiae semen, and *Rosa rugosa* Thunb.) were combined at a fixed ratio of 6:5:5:4:1 and boiled twice in water to obtain the extract. The combined extracts were concentrated and then vacuum-dried. This extraction method was designed to replicate the profile of bioactive compounds extracted under routine tea-drinking conditions, while maintaining the safety of components in RLMCR. To ensure batch-to-batch consistency, all raw materials were sourced from identical producing regions and complied with herbal pharmacopoeia standards, and unified extraction protocols were implemented across all batches. Rubusoside and nuciferine were quantified and defined as representative quality-control markers.

Antibodies specific for UCP1 were purchased from Santa Cruz Biotechnology, Inc. (California, USA); those for PGC-1α were obtained from Affinity Biosciences (Jiangsu, China), and those for *β*-ACTIN were purchased from Signalway Antibody (Greenbelt, USA). Vancomycin, neomycin sulfate, metronidazole, and ampicillin were purchased from Abmole Bioscience Inc. (Houston, USA). The high-fat diet (Catalog No. D12492) was obtained from Research Diets, Inc. (New Brunswick, USA), and contained 20% protein, 20% carbohydrates, and 60% fat.

### 2.2 Animal experiment

#### 2.2.1 Animals

Seven-week-old male C57BL/6J mice weighing 20 ± 2 g were obtained from the Laboratory Animal Center of Central South University. All animals were housed under specific pathogen-free conditions at a controlled temperature of 24 ± 2 °C with a 12-h light/dark cycle. Mice were provided with food and water ad libitum. After 1 week of acclimatization, the animals were subjected to experimentation.

All procedures involving animals were performed in accordance with the Guide for the Care and Use of Laboratory Animals and were approved by the Institutional Animal Care and Use Committee of Central South University (Ethical Committee Approval Code: CSU-2024-0146).

#### 2.2.2 RLMCR administration

Male C57BL/6J mice were randomly divided into three groups (*n* = 10): control diet (CD) group, Vehicle group, and RLMCR group. Mice in the CD group were fed a control diet, while the remaining mice were fed a high-fat diet. To ensure precise control of the administered dosage, intragastric gavage was adopted as the delivery method. The RLMCR group was treated with 3.0 g/kg RLMCR daily by oral gavage for 8 weeks, whereas other groups received an equivalent volume of sterile water.

The dose of 3.0 g/kg/day was determined from our previous dose-finding study in diet-induced obese mice, which evaluated three doses (1.5, 3.0, and 4.5 g/kg/day) and identified the middle dose (3.0 g/kg/day) as the optimal dose for achieving weight loss effects (3). Dose conversion was performed following the standard body surface area (BSA) conversion formula for interspecies translation. Assuming an adult human body weight of 70 kg, the daily human-equivalent intake of RLMCR was calculated as: 70 kg × 0.081 (the conversion coefficient between humans and mice) × 3.0 g/kg = 17 g RLMCR per day (14). This calculated dosage provides a reference for subsequent dietary intervention in humans. A positive control was deliberately omitted in this study. First, to our knowledge, no currently available multi-target agent exists for the prevention of obesity and its metabolic complications that would be directly comparable to RLMCR. Second, our aim was not to position RLMCR as an equivalent to weight-loss pharmacotherapy, but rather to evaluate its potential as a lifestyle intervention to aid in obesity management.

#### 2.2.3 Fecal microbiota transplantation experiment

##### 2.2.3.1 Donor mice and fecal harvesting

To guarantee the efficacy of fecal microbiota transplantation, fresh mouse feces were collected daily and transplanted on the same day without freezing. Under specific pathogen-free conditions, fresh fecal samples were obtained from mice in the RLMCR and vehicle groups. For sample pooling, all fecal pellets harvested from mice within the same group were combined into a single EP tube. Subsequently, 100 mg of feces were homogenized and diluted with 1 mL sterile PBS, followed by centrifugation at 600 rpm for 5 min (15).

##### 2.2.3.2 Recipient mice

HFD-fed male C57BL/6J mice were randomly divided into two groups (*n* = 10): vehicle-FMT group and RLMCR-FMT group. To eliminate interference from endogenous gut microbiota, recipient mice received a daily oral gavage of an antibiotic cocktail for 1 week, composed of vancomycin (100 mg/kg), neomycin sulfate (200 mg/kg), metronidazole (200 mg/kg), and ampicillin (200 mg/kg). Subsequently, mice in the vehicle-FMT group were orally gavaged with 0.1 mL of bacterial fluid obtained from vehicle group donors, and the RLMCR-FMT group received 0.1 mL of bacterial fluid from RLMCR group donors once daily for 12 weeks. Moreover, fecal anaerobic culture was implemented to validate antibiotic-mediated microbiota depletion and successful FMT. Higher bacterial colony counts were detected on agar plates after transplantation, confirming successful microbial engraftment (Supplementary Figure 1).

### 2.3 Glucose metabolism assessment

For the intraperitoneal glucose tolerance test (IPGTT), mice were fasted for 16 h and then injected intraperitoneally with glucose at 2.0 g/kg. For the insulin tolerance test (ITT), mice were fasted for 4 h and injected intraperitoneally with insulin at 0.75 U/kg. Blood glucose levels were measured before injection and at 15, 30, 60, 90, and 120 min post-injection.

### 2.4 Body composition analysis

At the end of the intervention, the body composition of mice, including fat mass and lean mass, was quantitatively determined using a time-domain nuclear magnetic resonance (TD-NMR) body composition analyzer (Bruker MiniSpec LF 50, Germany).

### 2.5 Energy expenditure

Whole-body energy expenditure, food intake, physical activity, and thermogenesis were monitored over a 72-h period using a Comprehensive Laboratory Animal Monitoring System (Columbus Instruments, USA). Mice were housed individually under a 12-h light/dark cycle at room temperature with ad libitum access to food and water throughout the monitoring period. All energy expenditure parameters, including oxygen consumption, carbon dioxide production, and heat production, were normalized to body weight.

### 2.6 Biochemical parameter analysis

After the intervention, glycosylated hemoglobin (HbA1c) levels were measured according to the manufacturer’s instructions (Sinocare Inc., China). For serum collection, mice were fasted overnight and anesthetized by inhalation of 2% isoflurane at a fresh gas flow rate of 4 L/min. Blood was collected from the retro-orbital sinus. All animal handling and euthanasia procedures were performed in accordance with institutional animal care guidelines. The blood was allowed to clot at room temperature for 2 h and then centrifuged at 3,500 rpm for 10 min. The supernatant (serum) was collected and stored at −80 °C until use. Concentrations of glucose, alanine aminotransferase (ALT), aspartate transaminase (AST), and triglycerides (TG) were measured in the Department of Laboratory Medicine, The Second Xiangya Hospital, using routine diagnostic methods.

### 2.7 Histological analysis

Liver, epididymal adipose tissue, inguinal adipose tissue, and brown adipose tissue were collected from euthanized mice for histological analysis. Tissue samples were fixed in 10% neutral buffered formalin, embedded in paraffin, and sectioned. Hematoxylin and eosin (H&E) staining was performed in accordance with the manufacturer’s instructions (Beyotime, China). Stained sections were visualized, and images were captured using a light microscope (Olympus, Japan).

### 2.8 Cold exposure and temperature measurements

For cold tolerance assessment, mice were housed individually. Baseline core temperature was measured by recording rectal temperature at room temperature. Subsequently, mice were placed in a 4 °C environment, and rectal temperature was recorded hourly over a 6-h period.

### 2.9 16S rDNA gene sequencing of the fecal microbiota composition

Fecal samples were collected from each group of mice and sent to Biotree BIOTECH Co., Ltd. (Shanghai, China) for gut microbiota analysis. Total fecal microbial DNA was obtained from fecal samples using the Fecal Genome DNA Extraction Kit (BioTeke, China) and quantified with a Qubit fluorometer (Invitrogen, USA). The 16S rDNA gene was amplified by PCR, and the amplified products were purified and subjected to paired-end sequencing (2 × 250 bp) on an Illumina NovaSeq 6000 platform.

Raw sequencing data were processed using the QIIME2 pipeline. Alpha and beta diversity metrics were calculated with QIIME2, and bacterial taxonomic profiles were generated based on relative abundance. Differentially abundant genera between groups were identified using the Wilcoxon rank-sum test, with statistical significance set at *p* < 0.05. LDA effect size (LEfSe, LDA ≥ 3.0, *p* < 0.05) was performed using nsegata-lefse. Other diagrams were generated using R software (v3.4.4).

### 2.10 Fecal untargeted metabolomics

Fecal samples were extracted using a mixed solvent of methanol, acetonitrile, and water. After homogenization and sonication in an ice-cooled water bath, samples were centrifuged at 12,000 rpm for 15 min. The supernatants were collected into fresh glass vials before LC–MS/MS analysis. Polar metabolites were separated on a Vanquish UHPLC system (Thermo Fisher Scientific) fitted with a Waters ACQUITY UPLC BEH Amide column (2.1 mm × 50 mm, 1.7 μm), which was interfaced with an Orbitrap Exploris 120 mass spectrometer (Thermo). Mobile phase A consisted of aqueous 25 mmol/L ammonium acetate and 25 mmol/L ammonium hydroxide (pH = 9.75), while mobile phase B was acetonitrile. The autosampler was held at 4 °C, and the injection volume was fixed at 2 μL. Mass spectral data were acquired using an Orbitrap Exploris 120 mass spectrometer operated in the information-dependent acquisition (IDA) mode via Xcalibur software, enabling continuous evaluation of full-scan MS spectra and automated MS/MS spectral acquisition. Raw MS data were converted to mzXML format using ProteoWizard and processed via an in-house R program based on XCMS for peak detection, extraction, alignment, and integration. Metabolite identification was performed using R packages and BiotreeDB (V3.0).

### 2.11 Quantitative real-time PCR

Tissue samples were homogenized in TRIzol reagent (Thermo Fisher Scientific, USA) for total RNA extraction according to the manufacturer’s protocol. Reverse transcription was carried out using a PrimeScript™ RT kit (TaKaRa, Japan) to generate cDNA. Quantitative real-time PCR amplification was performed on a LightCycler 480 system (Roche, Switzerland) with SYBR Green master mix (TaKaRa, Japan). The primers used are listed in Table 1. Target gene mRNA levels were normalized to *β-actin* and expressed as fold change relative to controls using the 2^−ΔΔCT^ method.

**Table 1:** Sequences of primers for qRT-PCR analysis.

Gene	Forward primer (5′ to 3′)	Reverse primer (5′ to 3′)
*Actin*	CAACGAGCGGTTCCGATG	GCCACAGGATTCCATACCCA
*Ucp1*	AGGCTTCCAGTACCATTAGGT	CTGAGTGAGGCAAAGCTGATTT
*Pgc-1α*	CCCTGCCATTGTTAAGACC	TGCTGCTGTTCCTGTTTTC
*Prdm16*	CTTAGCCGGGAAGTCACAGG	CCTCAACACACCTCCGGGTA
*Cidea*	AAAGGGACAGAAATGGACACC	TACATCGTGGCTTTGACATTG
*Dio2*	CTCCTAGATGCCTACAAACAGGTTA	GTCAAGAAGGTGGCATTCGG

### 2.12 Western blotting

Total protein was extracted with the radio immunoprecipitation assay (RIPA) lysis buffer and quantified using the bicinchoninic acid (BCA) assay. Equal amounts of protein were loaded and separated by SDS-PAGE gel, then transferred onto activated PVDF membranes (Millipore, USA). After blocking with 5% skimmed milk for 1 to 2 h, membranes were incubated with primary antibodies overnight, followed by secondary antibody incubation for 1 h. Protein bands were visualized using enhanced chemiluminescence (ECL) and quantified by ImageJ.

### 2.13 Statistical analysis

Statistical analysis was carried out using SPSS 27.0. Data are expressed as mean ± the standard error mean (SEM). Group comparisons were evaluated by one-way analysis of variance (ANOVA), Student’s *t*-test, Mann–Whitney U test, or chi-square test as appropriate. *p*-value < 0.05 was considered a significant difference, with marked as *p < 0.05, **p < 0.01, ***p < 0.001, and ****p < 0.0001; *p*-value > 0.05 was designated as ns (no significant difference). Correlations between variables were assessed using Spearman’s rank test. Graphs were generated using GraphPad Prism 9 software.

## 3 Results

### 3.1 RLMCR prevented HFD-induced glucolipid metabolic disorders in mice

**Figure 1:**
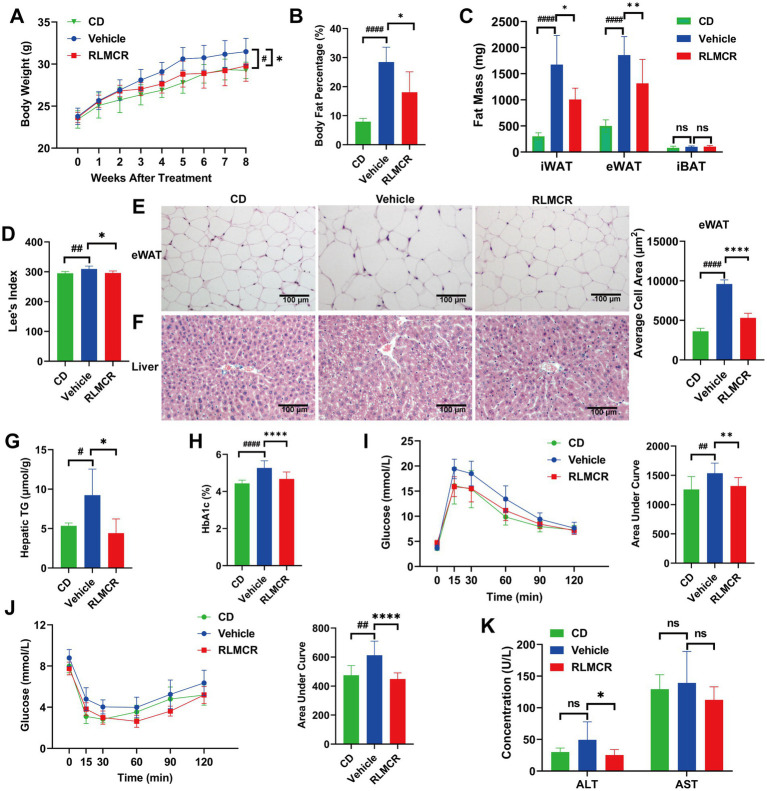
RLMCR alleviated weight gain and glucolipid metabolic disorders in HFD-fed mice. **(A)** Weekly body weight of mice throughout the 8-week intervention. **(B)** Body fat percentage. **(C)** Fat mass of different adipose depots. **(D)** Lee’s index. Representative H&E staining of **(E)** eWAT and **(F)** livers (scale bar = 100 μm). **(G)** Hepatic triglyceride content. **(H)** HbA1c level. **(I)** Glucose tolerance test (GTT) and area under the curve (AUC). **(J)** Insulin tolerance test (ITT) and AUC. **(K)** Concentrations of serum AST and ALT. Data are presented as mean ± SEM. *p < 0.05, **p < 0.01, ****p < 0.0001 RLMCR group vs. vehicle group; #*p* < 0.05, ##*p* < 0.01, ####*p* < 0.0001 CD group vs. vehicle group; ns: not significant (*n* = 10).

To evaluate the preventive effect of RLMCR against HFD-induced obesity and associated metabolic disorders, C57BL/6J mice were orally administered RLMCR at a dose of 3.0 g/kg/day concurrently with HFD feeding. This dosage was determined based on prior dose-optimization studies in DIO mice, which confirmed 3.0 g/kg/day as the most effective dose for weight loss. After 8 weeks of intervention, our results showed that RLMCR alleviated HFD-induced body weight gain and reduced the body fat percentage in mice (Figures 1A,B). The weight changes of different adipose tissue depots were further measured; RLMCR significantly decreased epididymal white adipose tissue (eWAT) and inguinal white adipose tissue (iWAT) mass (Figure 1C). Compared with the vehicle control group, Lee’s index—an indicator of obesity—was significantly lower after RLMCR intervention (Figure 1D). H&E staining of eWAT revealed that RLMCR reduced adipocyte size in visceral fat and attenuated intracellular lipid droplet accumulation (Figure 1E). RLMCR intervention also led to a marked decrease in liver triglyceride (TG) content and alleviated hepatic steatosis relative to the vehicle control (Figures 1F,G). Meanwhile, glucose metabolism in HFD-fed mice was effectively improved by RLMCR, which significantly lowered HbA1c, enhanced glucose tolerance, and elevated insulin sensitivity (Figures 1H–J). Notably, serum ALT, a marker of hepatocellular injury, was markedly lower in the RLMCR group, suggesting that RLMCR is not only safe but may also ameliorate diet-induced liver injury (Figure 1K). Collectively, these findings demonstrated that RLMCR exerted a significant preventive effect against diverse HFD-induced metabolic abnormalities.

### 3.2 RLMCR increased energy expenditure by promoting WAT browning

**Figure 2:**
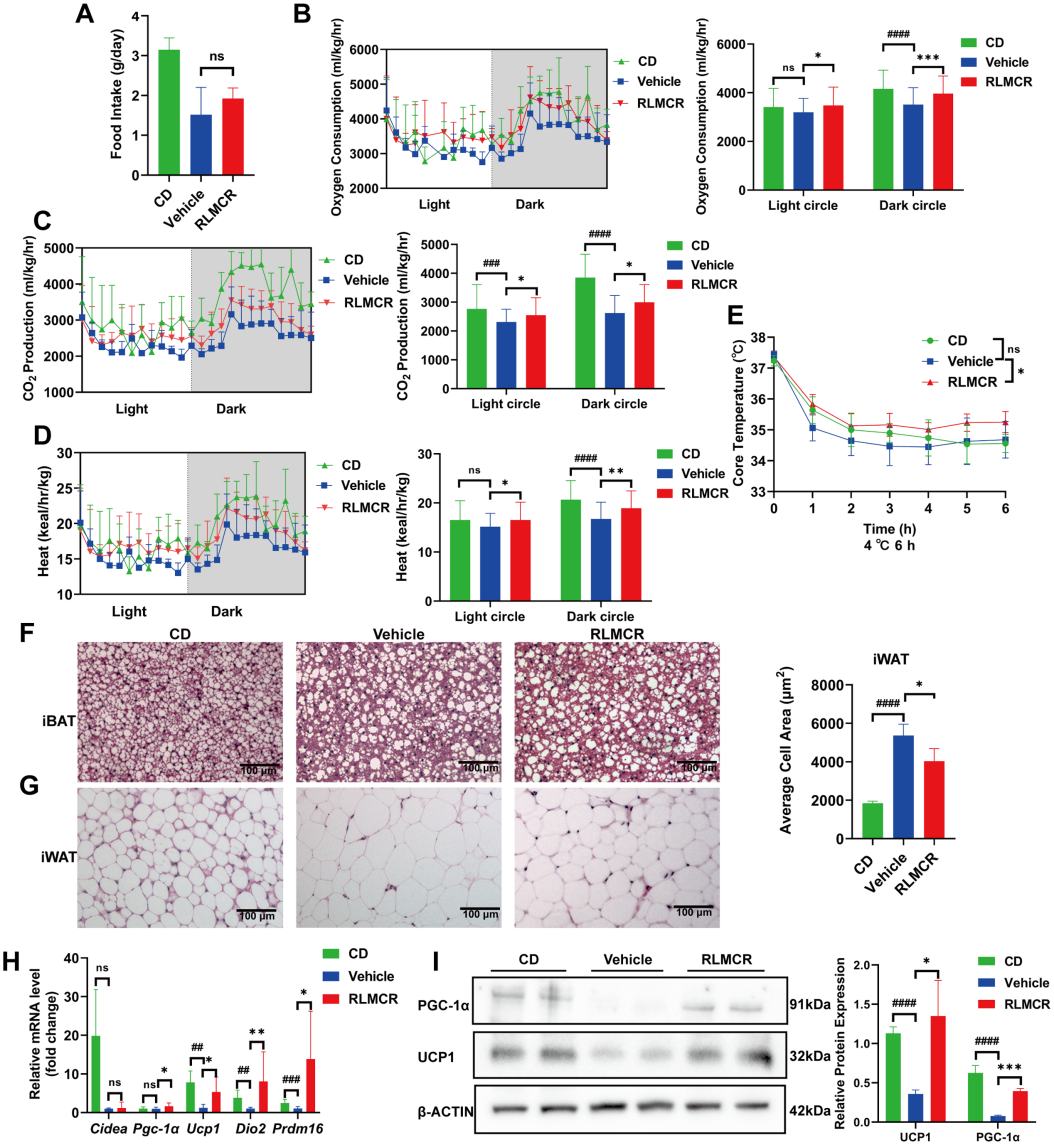
RLMCR enhanced energy metabolism, stimulated thermogenesis, and promoted WAT browning in HFD-fed mice. **(A)** Food intake **(B)** oxygen consumption, **(C)** carbon dioxide production, and **(D)** heat production over 24 h (*n* = 6). **(E)** Changes in core body temperature during cold exposure (*n* = 4). Representative H&E staining of **(F)** BAT and **(G)** iWAT (Scale bar = 100 μm). **(H)** mRNA expression of *Cidea*, *Pgc1-α*, *Ucp1*, *Dio2*, and *Prdm*16 in iWAT. **(I)** Protein expression of UCP1 and PGC1-α in iWAT. Data are presented as mean ± SEM. *p < 0.05, **p < 0.01, ***p < 0.001 RLMCR group vs. vehicle group; ##*p* < 0.01, ###*p* < 0.001, ####*p* < 0.0001 CD group vs. vehicle group; ns: not significant.

To assess the impact of RLMCR on energy metabolism, metabolic cage analysis was performed on mice from the CD, vehicle, and RLMCR groups. RLMCR intervention did not affect 24-h food intake compared with the vehicle group, whereas oxygen consumption, carbon dioxide production, and heat production were significantly increased in RLMCR-treated mice (Figures 2A–D). These results are consistent with the phenotype we previously observed in DIO mice (3), indicating that RLMCR may exert its effects by enhancing energy metabolism without altering food intake. Adipose tissue thermogenesis is widely recognized as a critical component of whole-body energy metabolism and is involved in the maintenance of core body temperature under cold exposure. To examine the effect of RLMCR on this process, mice from each group were placed in a 4 °C environment, and rectal temperature was measured hourly. Core temperature was found to be significantly higher in the RLMCR group than in the vehicle group, indicating that RLMCR intervention enhanced cold tolerance (Figure 2E). Intrascapular brown adipose tissue (iBAT), a major thermogenic organ, was first examined, but no morphological changes were observed after RLMCR treatment (Figure 2F). In addition to BAT, white adipose tissue (WAT) also contributes to cold adaptation via browning—a process characterized by upregulated thermogenic genes, increased mitochondria, and multilocular lipid droplet formation. As the most browning-prone depot, iWAT was chosen for further analysis. To determine whether RLMCR promoted the browning of iWAT in HFD-fed mice, H&E staining was first performed to evaluate morphological changes. Compared with the vehicle group, RLMCR-treated mice exhibited reduced adipocyte size and an increased number of multilocular cells in iWAT (Figure 2G). The expression levels of thermogenesis-related genes in iWAT were subsequently examined. RLMCR significantly upregulated the mRNA levels of *Pgc-1α* (peroxisome proliferator-activated receptor *γ* coactivator 1-α), *Ucp1* (Uncoupling protein 1), *Dio2* (iodothyronine deiodinase type II), and *Prdm16* (PR domain-containing 16) in iWAT, while *Cidea* (cell death inducing DFFA like effector A) tended to increase (Figure 2H). Given the central roles of UCP1—an inner mitochondrial membrane protein that uncouples oxidative phosphorylation to dissipate energy as heat (16)—and PGC-1α—a transcriptional coactivator that drives mitochondrial biogenesis and activates thermogenic genes (17), we further evaluated the protein expression levels of these two molecules. Western blot analysis confirmed that RLMCR markedly elevated the protein levels of UCP1 and PGC-1α in the iWAT of HFD-fed mice (Figure 2I). Protein expression levels of UCP1 and PGC-1α in iBAT were also detected, with no significant alterations observed following RLMCR treatment (Supplementary Figure 2), which further confirms that RLMCR-induced thermogenic activation is independent of brown adipose tissue. The results above indicated that RLMCR increased whole-body energy expenditure by inducing WAT browning and enhancing its thermogenic capacity.

### 3.3 RLMCR ameliorated HFD-induced gut microbiota dysbiosis

**Figure 3:**
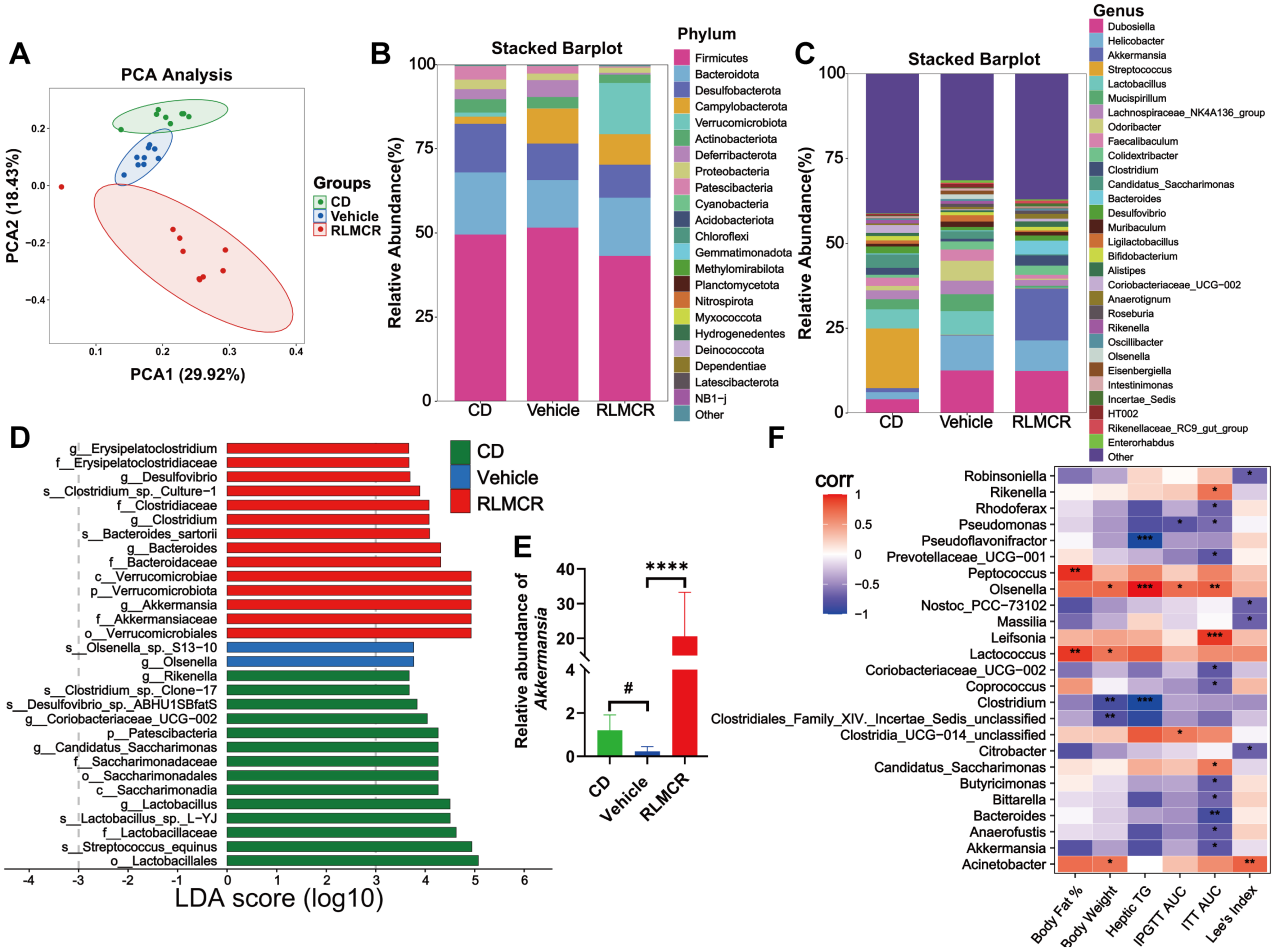
RLMCR regulated the composition of gut microbiota in HFD-fed mice. **(A)** Principal coordinates analysis (PCoA) of gut microbiota composition. **(B)** Relative abundance of gut microbiota at the phylum level. **(C)** Relative abundance of gut microbiota at the genus level. **(D)** Linear discriminant analysis effect size (LEfSe) cladogram of differentially abundant bacterial taxa. **(E)** Relative abundance of the genus *Akkermansia* (*n* = 8). **(F)** Heatmap of Spearman correlation analysis between differentially abundant bacterial genera and metabolic parameters (*n* = 24). *RS*: Spearman’s rank correlation coefficient. ****p < 0.0001 RLMCR group vs. vehicle group; #*p* < 0.05 CD group vs. vehicle group.

To investigate the impact of RLMCR on the structure of gut microbiota in HFD-fed mice, fecal samples were collected after 8 weeks of intervention and subjected to 16S rDNA sequencing analysis. Principal component analysis (PCA) revealed significant differences in gut microbiota composition among the three groups, indicating that RLMCR significantly altered the microbial structure in HFD-fed mice (Figure 3A). Subsequently, the relative abundance of bacteria at the phylum level was compared between groups to further characterize gut microbiota composition. Compared with the CD group, the vehicle group showed a significant decrease in the abundance of *Bacteroidota* and *Verrucomicrobia*, and an increase in *Firmicutes* abundance, and these diet-induced microbial alterations were effectively reversed by RLMCR intervention (Figure 3B). At the genus level, compared with the CD group, HFD treatment resulted in a marked decrease in the relative abundances of *Akkermansia*, *Coriobacteriaceae_UCG−002*, and *Clostridium*, and an increase in the relative abundances of *Olsenella* (Figure 3C). These alterations were also reversed by RLMCR treatment. Linear discriminant analysis (LDA) effect size (LEfSe) analysis revealed that *Akkermansia muciniphila* (*A. muciniphila*) was the dominant microbiota in the RLMCR-treated group (Figure 3D). Zhang et al. demonstrated that *A. muciniphila* supplementation significantly reduced body weight and HbA1c levels in overweight/obese patients with type 2 diabetes (18). In our study, the relative abundance of the *Akkermansia* genus increased nearly 100-fold in the gut of HFD-fed mice following RLMCR intervention, suggesting that increasing the abundance of this probiotic may play an important role in the anti-obesity mechanism of RLMCR (Figure 3E).

To explore the potential association between gut microbiota and metabolic phenotypes, Spearman correlation analysis was conducted. At the genus level, most varying bacteria showed significant positive or negative correlations with measures of obesity, glucose homeostasis, and lipid metabolism (Figure 3F). Specifically, *Olsenella* abundance correlated positively with body weight (R_S_ = 0.73, *p* = 0.0158), hepatic triglycerides (R_S_ = 1, *p* < 0.0001), area under the curve (AUC) of ITT (R_S_ = 0.71, *p* = 0.0217), and IPGTT (R_S_ = 0.77, *p* = 0.0092). *Acinetobacter* correlated positively with body weight (R_S_ = 0.68, *p* = 0.0323) and Lee’s index (R_S_ = 0.77, *p* = 0.0077), while *Peptococcus* correlated positively with body fat percentage (R_S_ = 0.94, *p* = 0.0048). In contrast, *Pseudoflavonifractor* correlated negatively with hepatic triglycerides (R_S_ = −1, *p* < 0.0001), and *Akkermansia* correlated negatively with AUC of ITT (R_S_ = −0.73, *p* = 0.0158). Taken together, RLMCR markedly reshaped the gut microbiota structure in HFD-fed mice, with the most pronounced effect being the marked enrichment of *Akkermansia*.

### 3.4 RLMCR reshaped gut metabolite profiles in HFD-fed mice

**Figure 4:**
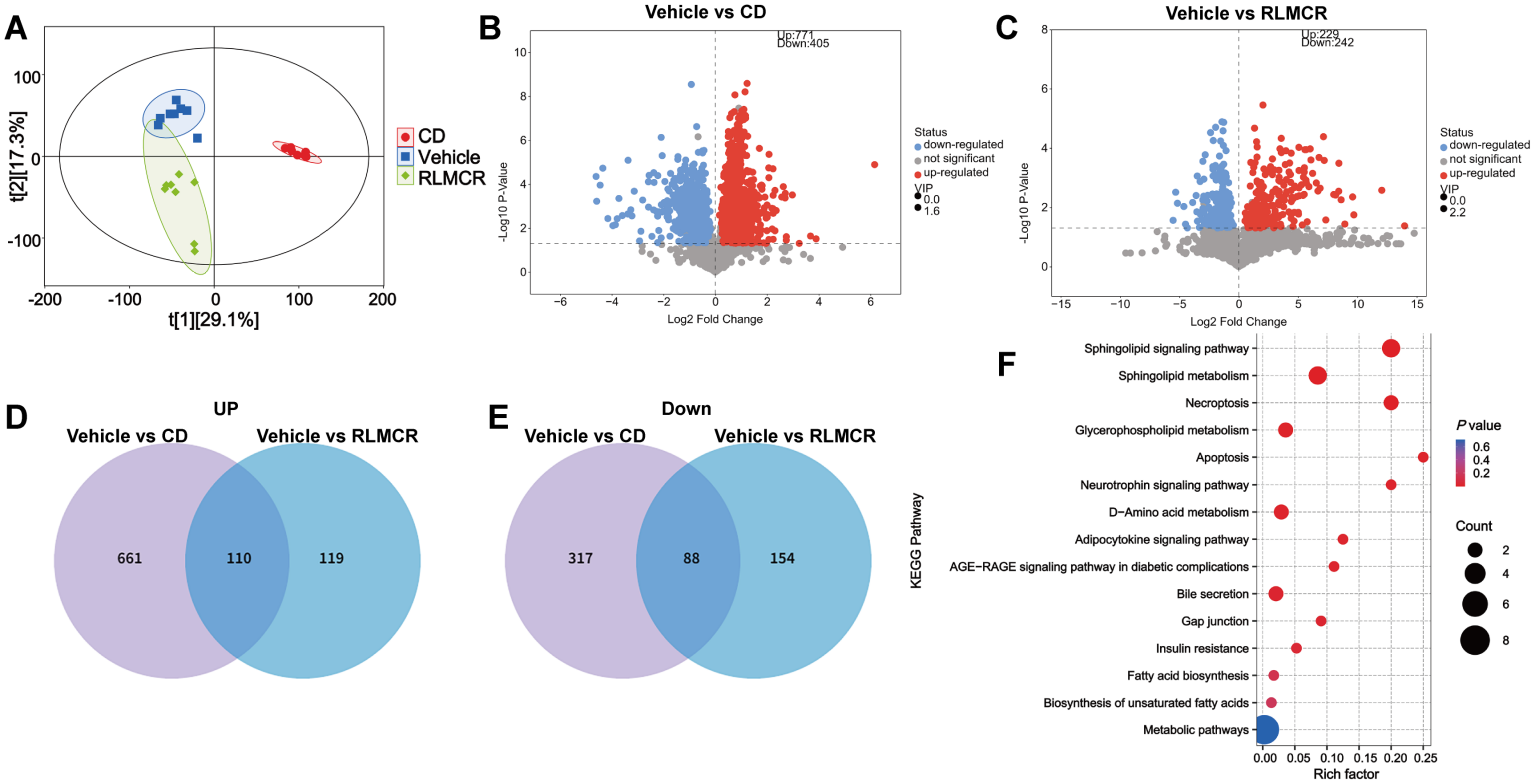
Effects of RLMCR on gut metabolites in HFD-fed mice. **(A)** Partial least squares discriminant analysis (PLS-DA) score plot of fecal metabolites. **(B)** Volcano plot of Vehicle vs. CD differential metabolites. **(C)** Volcano plot of Vehicle vs. RLMCR differential metabolites. **(D)** Venn diagram showing the intersection of metabolites up-regulated in the CD group compared to the Vehicle group and those up-regulated in the RLMCR group compared to the Vehicle group. **(E)** Venn diagram showing the intersection of metabolites down-regulated in the CD group compared to the Vehicle group and those down-regulated in the RLMCR group compared to the Vehicle group. **(F)** KEGG pathway enrichment analysis. (*n* = 8).

To investigate the effects of RLMCR on gut metabolites, untargeted metabolomics analysis was conducted on fecal samples collected from mice in each group. Partial least squares discriminant analysis (PLS-DA) revealed that RLMCR markedly reshaped the gut metabolite profiles of HFD-fed mice (Figure 4A). Differential metabolites were screened based on the criteria of VIP > 1 and *p*-value < 0.05. Compared with the Vehicle group mice fed a high-fat diet, 771 metabolites were significantly upregulated, and 405 metabolites were downregulated in the CD group (Figure 4B). After RLMCR intervention, 229 metabolites were upregulated, and 242 metabolites were downregulated in HFD-fed mice (Figure 4C). We further calculated the intersection of differentially expressed metabolites with consistent trends in the CD and RLMCR groups relative to the Vehicle group. A total of 110 metabolites were synchronously upregulated, while 88 metabolites were concurrently downregulated in both groups (Figures 4D,E). Detailed information on these metabolites is listed in Supplementary Tables S1, S2. KEGG enrichment analysis was performed on the shared differential metabolites. Figure 4F displays the top 15 pathways that were most significantly impacted by RLMCR intervention, including sphingolipid signaling pathway, sphingolipid metabolism, glycerophospholipid metabolism, bile secretion, adipocytokine signaling pathway, and insulin resistance (*p* < 0.05). Consequently, the composition of gut metabolites was significantly altered by RLMCR in HFD-fed mice.

### 3.5 RLMCR alleviated HFD-induced obesity and metabolic disorders via the gut microbiota

**Figure 5:**
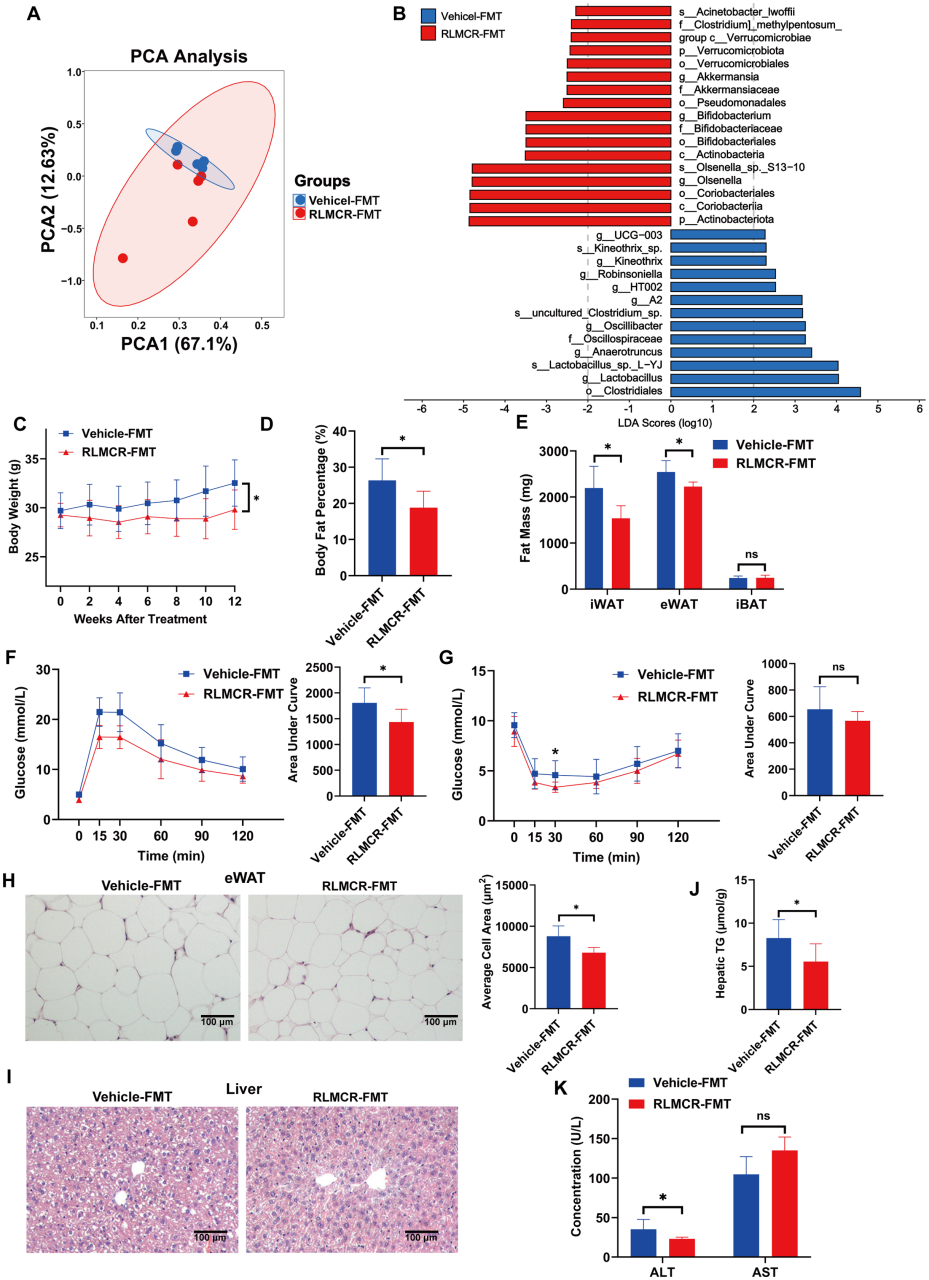
FMT ameliorated glucose and lipid metabolic disorders in HFD-fed mice. **(A)** Principal coordinates analysis (PCoA) of gut microbiota composition. **(B)** Linear discriminant analysis effect size (LEfSe) cladogram of differentially abundant bacterial taxa (*n* = 5). **(C)** Body weight. **(D)** Body fat percentage. **(E)** Fat mass of different adipose depots. **(F)** IPGTT results. **(G)** ITT results. Representative H&E staining of **(H)** eWAT and **(I)** livers (scale bar = 100 μm). **(J)** Hepatic triglyceride content. **(K)** Serum AST and ALT. Data are presented as mean ± SEM. ns: not significant, *p < 0.05, RLMCR-FMT group vs. vehicle-FMT group (*n* = 10).

To directly investigate whether the gut microbiota plays a crucial role in the anti-obesity effect of RLMCR, a fecal microbiota transplantation (FMT) experiment was performed. Gut microbiota from RLMCR and vehicle donor groups were transplanted into antibiotic-pretreated C57BL/6J recipient mice via oral gavage. 16S rDNA sequencing of fecal samples from FMT recipient mice showed distinct microbial profiles between the RLMCR-FMT group and the vehicle-FMT group (Figure 5A). Furthermore, consistent with the donor mice, LEfSe analysis further revealed that the relative abundance of the *Akkermansia* genus was significantly increased in the RLMCR-FMT group (Figure 5B). These results confirmed successful microbial engraftment. After 12 weeks of FMT, gut microbiota from the RLMCR group significantly alleviated HFD-induced body weight gain and fat accumulation in recipient mice, accompanied by significant reductions in eWAT and iWAT mass (Figures 5C–E). Additionally, IPGTT and ITT results showed that mice in the RLMCR-FMT group exhibited improved glucose tolerance and enhanced insulin sensitivity compared with the vehicle-FMT group (Figures 5F,G). Moreover, compared to the vehicle-FMT group, the RLMCR-FMT group exhibited less lipid droplet accumulation in both visceral adipose tissue and the liver (Figures 5H–J). Consistent with findings in donor mice, transplantation of gut microbiota from RLMCR-treated mice ameliorated HFD-induced liver injury, as evidenced by reduced serum ALT levels (Figure 5K). These results demonstrated that transferring the gut microbiota from RLMCR-intervened mice significantly alleviated diet-induced obesity and associated metabolic disturbances, confirming that the gut microbiota plays an essential mediating role in RLMCR’s therapeutic effects.

### 3.6 RLMCR-modulated gut microbiota increased energy expenditure by inducing WAT browning

**Figure 6:**
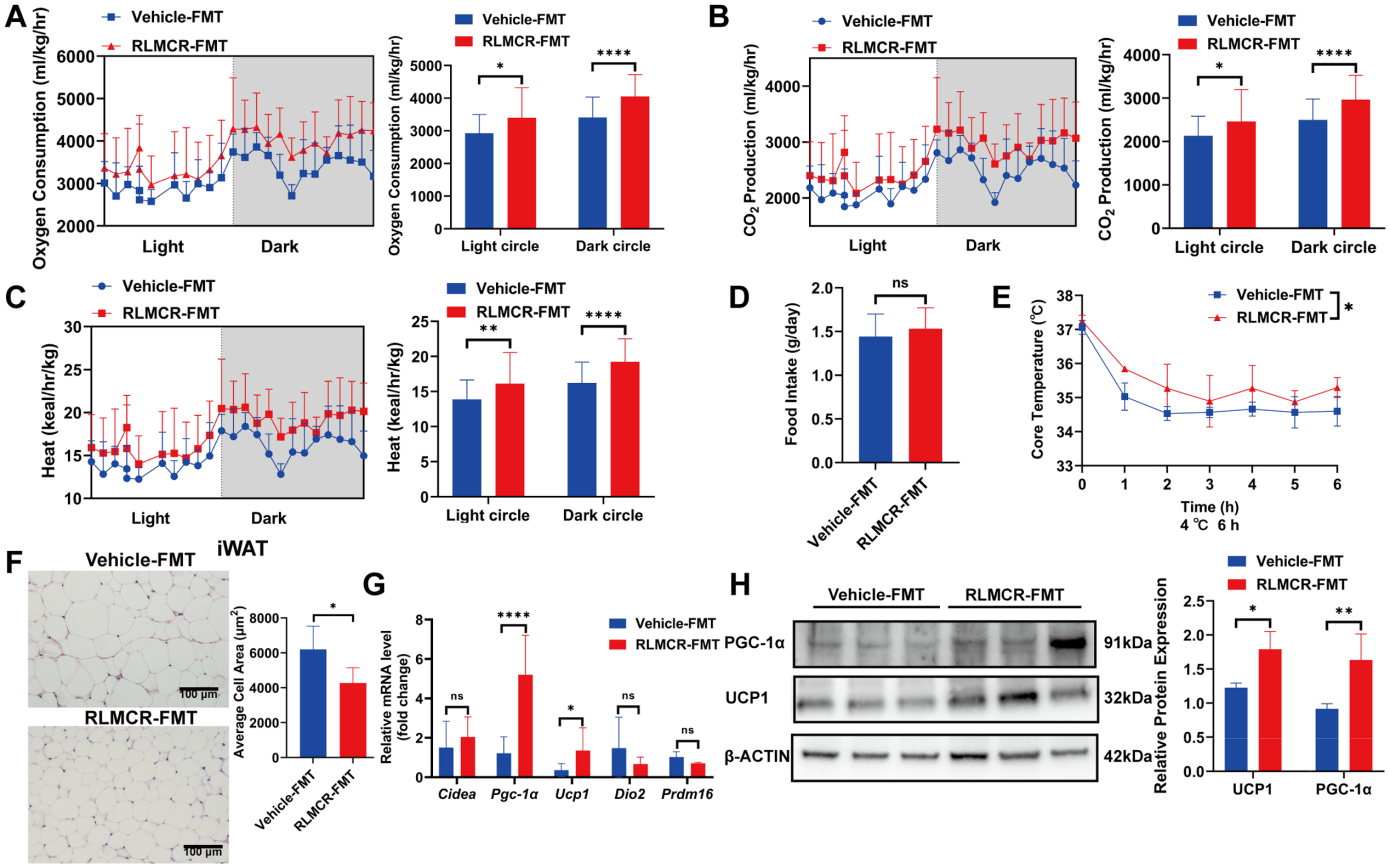
FMT increased energy expenditure and induced WAT browning in HFD-fed mice. **(A)** Oxygen consumption, **(B)** carbon dioxide production, **(C)** heat production, and **(D)** food intake over 24 h (*n* = 6). **(E)** Cold tolerance test. **(F)** Representative H&E staining of iWAT (Scale bar = 100 μm). **(G)** mRNA expression levels of *Cidea*, *Pgc1-α*, *Ucp1*, *Dio2*, and *Prdm*16 in iWAT. **(H)** Protein expression levels of UCP1 and PGC1-α in iWAT. Data are presented as mean ± SEM. ns: not significant, *p < 0.05, **p < 0.01, ****p < 0.0001 RLMCR-FMT group vs. vehicle-FMT group.

The gut microbiota serves as a crucial regulator of host energy metabolism, which significantly influences energy expenditure through its bioactive metabolites, including short-chain fatty acids (SCFAs) and bile acids. Consistently, untargeted metabolomics results indicated that differential metabolites modulated by RLMCR were also enriched in pathways related to bile secretion and lipid metabolism. To further investigate the role of gut microbiota in the RLMCR-mediated increase of energy expenditure, metabolic cage analysis was conducted in FMT recipient mice. The results showed that transplantation of microbiota from RLMCR-treated mice significantly increased oxygen consumption, carbon dioxide production, and heat generation in the recipient mice (Figures 6A–C). Meanwhile, there was no difference in food intake between the RLMCR-FMT group and the vehicle-FMT group (Figure 6D). These findings indicated that the gut microbiota mediated the enhancement of energy metabolism induced by RLMCR. To further examine whether the microbiota-driven increase in energy expenditure also occurs via enhanced thermogenesis and WAT browning, a cold tolerance test was performed. Core temperatures of mice in the RLMCR-FMT group were significantly higher than those of the vehicle-FMT group, demonstrating RLMCR-mediated gut microbiota enhanced thermogenesis (Figure 6E). Subsequently, morphological analysis of iWAT was carried out. The RLMCR-FMT group exhibited reduced lipid droplet size and an increased number of multilocular adipocytes in iWAT (Figure 6F). The expression levels of thermogenesis-related genes in iWAT from both groups were also examined. Transplantation of microbiota from RLMCR-treated mice significantly upregulated the expression of PGC-1α and UCP1 in iWAT (Figures 6G,H). Thus, the gut microbiota contributes to RLMCR-driven WAT browning.

## 4 Discussion

In recent years, obesity prevalence has risen sharply, imposing a substantial burden on individuals, families, and healthcare systems. Thus, robust interventions to prevent obesity onset are critical for public health. In the present study, we provide the first evidence that our self-developed patented solid drink RLMCR prevented HFD-induced obesity and its associated metabolic disturbances, offering a novel strategy for obesity prevention and control. Mechanistically, RLMCR exerted its effects by remodeling the gut microbiota, which in turn targeted iWAT to induce browning, stimulate thermogenesis, and enhance energy expenditure. Therefore, our findings suggest that RLMCR may exert comprehensive therapeutic effects through the “gut microbiota-adipose” axis, providing novel insights into obesity treatment.

Health maintenance relies on a dynamic balance within the gut microbiome between the “foundation guild” and “pathobiont guild.” The “foundation guild” supports host physiological functions (e.g., nutrient metabolism, immune regulation, energy homeostasis) by producing key metabolites such as short-chain fatty acids, vitamin K, and essential amino acids. Conversely, the “pathobiont guild” promotes disease development and consists primarily of opportunistic pathogens with antibiotic resistance genes and virulence factors. Once the seesaw-like equilibrium between these two competing guilds breaks down, systemic low-grade inflammation and various metabolic disorders like obesity and type 2 diabetes may develop. Targeted restoration of gut microbiota homeostasis offers a promising strategy for health maintenance. Among available interventions, dietary approaches stand out for their superior safety and long-term compliance in chronic condition management, therefore delivering durable, sustained benefits for metabolic health and the intestinal microenvironment. A randomized controlled trial in type 2 diabetes patients showed that a high-fiber diet markedly increased “foundation guild” abundance, and this increase was inversely correlated with HbA1c, blood lipids, and body weight (19). Nowadays, dietary interventions that target the gut microbiota have become a major strategy for improving metabolic health. In the present study, RLMCR intervention significantly reshaped the gut microbiota composition in HFD-fed mice. The phyla *Firmicutes* and *Bacteroidetes* are well-documented to be closely associated with metabolic disorders, particularly obesity. Elevated *Firmicutes* and reduced *Bacteroidetes* abundances in obese mice and humans link *Firmicutes* to obesity and *Bacteroidetes* to weight loss (5, 20, 21). Consistent with previous studies, HFD-fed mice exhibited higher *Firmicutes* abundance and lower *Bacteroidetes* abundance compared with the CD group, and these alterations were effectively attenuated by RLMCR treatment. Furthermore, RLMCR also reshaped the gut microbial composition at the genus level in HFD-fed mice. A marked elevation in the relative abundance of *Clostridium* was observed following RLMCR treatment. *Clostridium* is well-documented to exert preventive effects against obesity and related metabolic diseases. Mechanistically, Charisse et al. demonstrated that the anti-obesity effects of *Clostridia* are attributed to the inhibition of intestinal lipid absorption via the downregulation of CD36 expression (22). Moreover, short-chain fatty acid production, strengthened intestinal barrier, and reduced chronic inflammation also contribute to *Clostridium*’s anti-obesity effects (23, 24). In addition, *Olsenella*, a genus enriched in obese individuals and positively correlated with lipid levels (25), was significantly reduced after RLMCR intervention. Remarkably, an approximately 100-fold increase in the relative abundance of *Akkermansia* was detected in the gut of mice treated with RLMCR compared with the vehicle control mice. *Akkermansia muciniphila* (*A. muciniphila*) is a mucin-degrading bacterium belonging to the phylum *Verrucomicrobia* that resides within the intestinal mucus layer. Its unique localization not only enables *A. muciniphila* to contribute to the maintenance of a healthy gut barrier but also allows it to communicate directly with host cells, thereby regulating host physiology and metabolic status. In recent years, *A. muciniphila* has garnered increasing attention, with accumulating evidence indicating an inverse correlation between its relative abundance and various metabolic disorders, including obesity, non-alcoholic fatty liver disease, and type 2 diabetes (26). Direct supplementation with *A. muciniphila* has been shown to effectively reverse HFD-induced metabolic disturbances, including obesity, insulin resistance, adipose inflammation, and metabolic endotoxemia (27). Based on these findings, *A. muciniphila* is now recognized as a representative next-generation probiotic, and increasing its relative abundance has emerged as a promising therapeutic target. For instance, enrichment of *A. muciniphila* is a pivotal factor in mediating the metabolic benefits of metformin (28). In the present study, RLMCR treatment markedly upregulated the relative abundance of *A. muciniphila*, and FMT from RLMCR-treated mice significantly ameliorated HFD-induced glucose and lipid metabolic disturbances, confirming that the gut microbiota plays a crucial role in RLMCR’s anti-obesity efficacy. Notably, a higher relative abundance of *A. muciniphila* was also observed in the RLMCR-FMT group, suggesting that the enrichment of *A. muciniphila* may be closely associated with the metabolic benefits conferred by RLMCR. Specifically, this RLMCR-induced enrichment of *A. muciniphila* is likely attributed to its bioactive constituents, nuciferine and 1-deoxynojirimycin (29, 30), which we previously identified in RLMCR.

The regulatory effects of the gut microbiota on host metabolism depend on its influence on energy homeostasis (31). By generating bioactive metabolites such as short-chain fatty acids and bile acids, the gut microbiota acts on the gut-adipose axis, enhancing the thermogenic activity of adipose tissues to boost energy expenditure and reverse obesity-related energy imbalance (32). Li et al. demonstrated that, compared with conventional mice, both antibiotic-treated and germ-free mice exhibited downregulated UCP1 expression in WAT, accompanied by impaired WAT browning under cold exposure; this compromised thermogenic capacity could be restored by supplementation with butyrate, a key metabolite of gut microbiota (33). Currently, numerous natural products have been demonstrated to alleviate HFD-induced obesity and glucose intolerance through the promotion of WAT browning via the gut-adipose axis (34, 35). Consistently, our results revealed that RLMCR intervention induced WAT browning in HFD-fed mice, while FMT from RLMCR-treated mice recapitulated this effect, thereby identifying the enhancement of WAT browning as a key mechanism underlying the microbiota-mediated anti-obesity efficacy of RLMCR. Furthermore, RLMCR triggered marked changes in intestinal metabolites related to bile secretion and lipid metabolism, indicating that gut microbiota-mediated WAT browning may be associated with microbial metabolites. As the genus most profoundly affected by RLMCR, *A. muciniphila* has been widely recognized as a critical regulator of WAT browning. Wang et al. reported that administration of *A. muciniphila* in HFD-fed mice alleviated glucolipid metabolic abnormalities and markedly upregulated the expression of thermogenic genes, including PGC-1α, PRDM16, and UCP1, in iWAT (36). The induction of WAT browning may be attributed to SCFAs produced by *A. muciniphila*, of which acetate and butyrate have been proven to enhance UCP1 expression in iWAT through the miR-378a-YY1 signaling pathway (37). Thus, RLMCR-induced WAT browning is likely associated with its enrichment of *A. muciniphila*.

Although this study is the first to demonstrate that the novel patented solid drink RLMCR exerts preventive effects against HFD-induced obesity and clarifies its underlying mechanisms from the perspective of the gut-adipose axis, certain limitations remain. As a multi-component formulation, RLMCR likely acts through multiple mechanistic pathways; thus, its comprehensive mode of action and the specific roles of individual bioactive components require further investigation. Although our results identify a close correlation between increased *Akkermansia* abundance and the metabolic advantages of RLMCR, direct causal evidence is lacking. Further experiments involving *Akkermansia* depletion or direct bacterial supplementation need to be conducted to confirm its unique role in mediating the metabolic benefits of RLMCR. In addition, future research is required to identify the specific metabolites that modulate the gut-adipose axis through targeted quantification of fecal and serum microbial metabolites and verification of their associated signaling pathways. Apart from this, while we optimized the ingredients and water extraction procedure to secure the safety of RLMCR, systematic acute and chronic toxicity tests remain necessary to fully guarantee its safety in subsequent clinical translation. Moreover, subsequent multi-center, large-scale randomized controlled clinical trials in obese populations are needed to further evaluate RLMCR’s efficacy and optimize the intervention protocol, thereby providing more precise guidance for its clinical application. Finally, only a single dose of RLMCR was used in the present study; further investigations into the dose-dependent effects of RLMCR on gut microbiota and thermogenic markers are necessary in future work.

## 5 Conclusion

In conclusion, this study provides the first evidence that the novel patented solid drink RLMCR effectively prevents HFD-induced obesity and metabolic disorders, and modulates the gut microbiota with a marked enrichment of *A. muciniphila*. Mechanistically, our observations demonstrate that RLMCR exerts its anti-obesity effects via the gut-adipose axis by regulating the gut microbiota to induce WAT browning. These findings support RLMCR as a promising dietary intervention with potential for translational application in obesity management.

## Data Availability

The datasets presented in this study can be found in online repositories. The names of the repository/repositories and accession number(s) can be found at: https://www.ncbi.nlm.nih.gov/, PRJNA1435803.

## Glossary

ALT: alanine aminotransferase
AST: aspartate transaminase
AUC: area under the curve
*A. muciniphila*: *Akkermansia muciniphila*
BAT: brown adipose tissue
CD: control diet
DIO: diet-induced obese
Dio2: iodothyronine deiodinase type II
eWAT: epididymal white adipose tissue
FMT: fecal microbiota transplantation
HbA1c: glycosylated hemoglobin
HFD: high-fat diet
H&E: hematoxylin and eosin
IPGTT: intraperitoneal glucose tolerance test
ITT: insulin tolerance test
iWAT: inguinal white adipose tissue
LC–MS/MS: liquid chromatography–tandem mass spectrometry
PGC-1*α*: peroxisome proliferator-activated receptor *γ* coactivator 1-α
Prdm16: PR domain-containing 16
TG: triglycerides
UCP1: uncoupling protein 1
WAT: white adipose tissue.
