# Supplementary material for: A novel solid beverage RLMCR prevents obesity-related metabolic disorders through the gut-adipose axis
Source: Front Nutr. 2026 Jul 30;13:1895261. doi: 10.3389/fnut.2026.1895261 (PMC13468892; doi:10.3389/fnut.2026.1895261)

Supplementary material

Figure legends

Supplementary Figure 1. Verification of gut microbiota depletion and microbiota reconstitution in mice. (A) Fecal microbiota culture of mice after antibiotic treatment and fecal microbiota transplantation.

Supplementary Figure 2. RLMCR did not affect thermogenic gene expression in iBAT of HFD-fed mice. (A) Protein expression of UCP1 and PGC1-α in iBAT (n = 3). Data are presented as mean ± SEM. RLMCR group vs. vehicle group. ns: not significant.

Figures

Supplementary Figure 1.

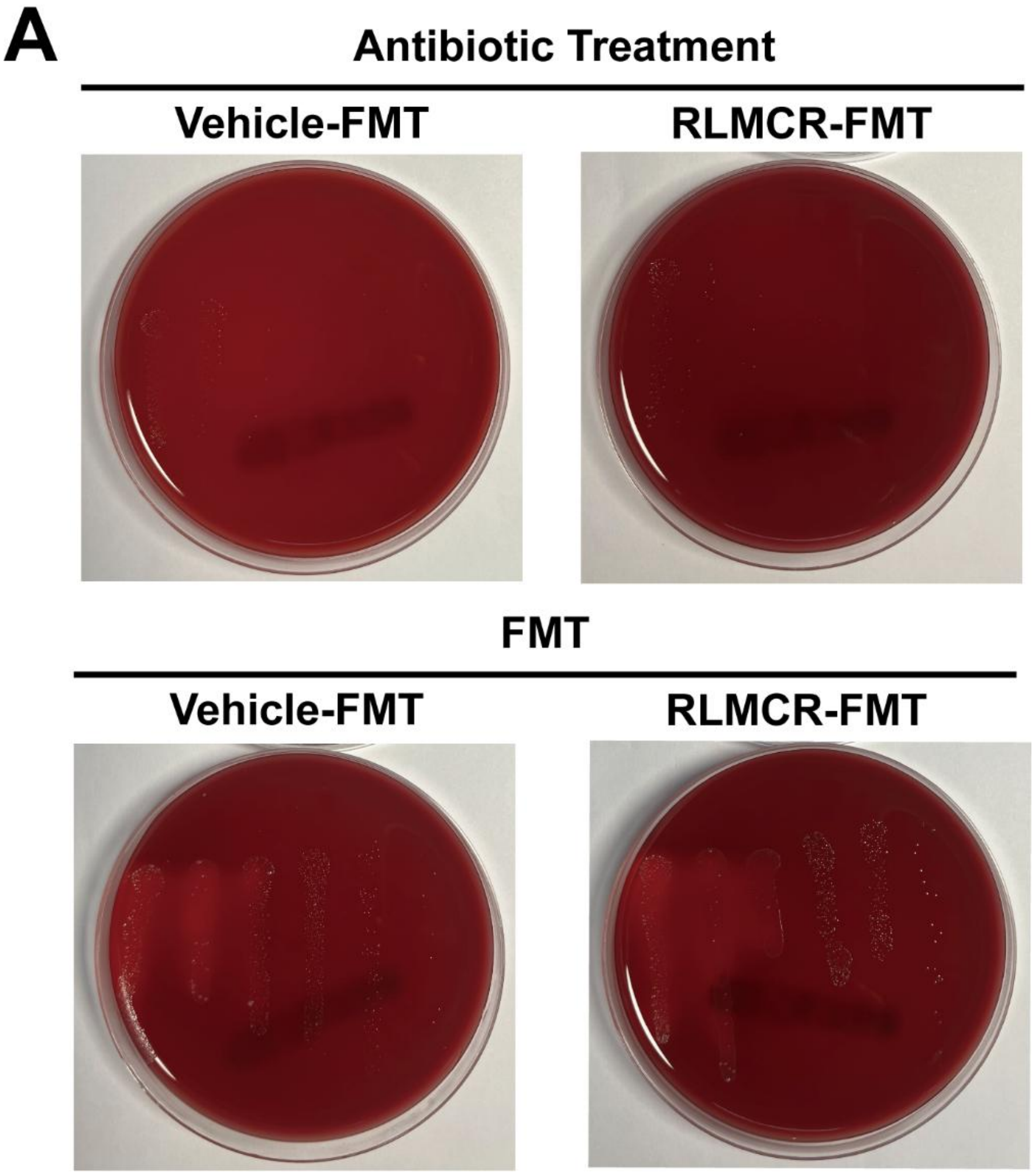

Supplementary Figure 2.

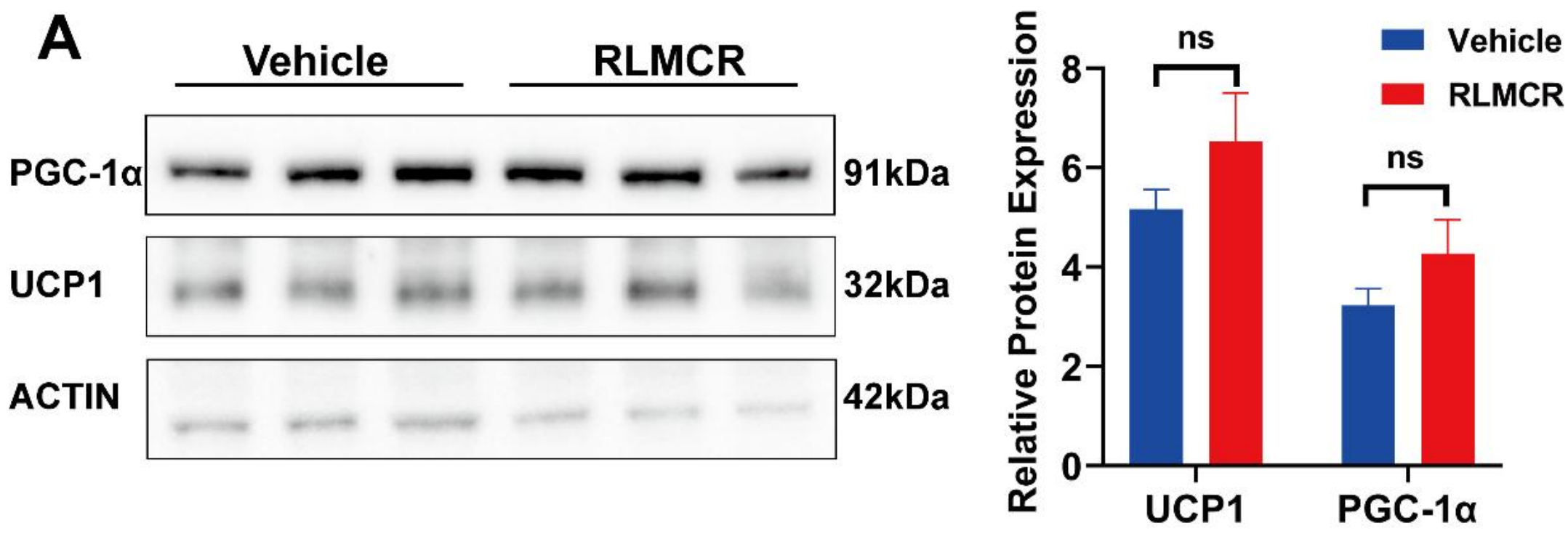

Supplement: Supplementary file 1 [file Data_sheet_1.pdf]
